# Complete Genome Sequence of the African Strain AXO1947 of *Xanthomonas oryzae* pv. oryzae

**DOI:** 10.1128/genomeA.01730-15

**Published:** 2016-02-11

**Authors:** J. C. Huguet-Tapia, Z. Peng, B. Yang, Z. Yin, S. Liu, F. F. White

**Affiliations:** a Department of Plant Pathology, University of Florida, Gainesville, Florida, USA; b Department of Plant Pathology, Kansas State University, Manhattan, Kansas, USA; c Department of Genetics, Development and Cell Biology, Iowa State University, Ames, Iowa, USA; d Temasek Life Sciences Laboratory, National University of Singapore, Singapore, Republic of Singapore

## Abstract

*Xanthomonas oryzae* pv. oryzae is the etiological agent of bacterial rice blight. Three distinct clades of *X. oryzae* pv. oryzae are known. We present the complete annotated genome of the African clade strain AXO194 using long-read single-molecule PacBio sequencing technology. The genome comprises a single chromosome of 4,674,975 bp and encodes for nine transcriptional activator-like (TAL) effectors. The approach and data presented in this announcement provide information for complex bacterial genome organization and the discovery of new virulence effectors, and they facilitate target characterization of TAL effectors.

## GENOME ANNOUNCEMENT

Bacterial blight of rice, caused by the Gram-negative bacterium *Xanthomonas oryzae* pv. oryzae, is the most important bacterial disease of rice and, possibly, the most important bacterial disease in terms of agronomic impact. Three clades of related bacteria have been classified as *X. oryzae* pv. oryzae, and complete genome sequences have been generated for Asian-related strains (1–3). Draft sequences are available in GenBank for several African linage strains, while one draft sequence is available for a North American strain (4). Strain AXO1947 (CFBP 1947) is of African origin (5)

Whole-genome sequencing of strain *X. oryzae* pv. oryzae AXO1947 was performed on the four single-molecule real-time cells of PacBio RS II. A total of 235,510 long circular reads (213-fold coverage) with an average length of 4,524 bp and 80% estimated accuracy were used as input for the self-correction and an assembly pipeline implemented in the PBcR package (6). The resulting contig was corrected by remapping reads with pbalign version 0.2, and a consensus was obtained with Quiver (7, 8). Final correction was performed for small insertions or deletions that were identified by mapping Illumina 2 × 300-bp MiSeq reads using Bowtie2 (9) with an average coverage of 88×. Error calling and generation of the final corrected assembly was conducted using the variant detection tool Pilon (10). Prediction and annotation of coding sequences were conducted with NCBI Prokaryotic Genome Annotation Pipeline (PGAAP) (11). Whole chromosome alignment and comparison was conducted with MUMmer3 (12).

The genome of *X. oryzae* pv. oryzae AXO1947 consists of a chromosome of 4,674,975 bp with 63.89% GC content. Preliminary draft annotation with PGAAP indicates that the genome is predicted to contain 3,706 genes encoding for proteins and 54 for tRNAs. Reciprocal average nucleotide identity (ANI index) (13) between AXO1947 and two other previously sequenced strains, POX99^A^ and MAFF311018, reveals that AXO1947 shares an average of 97% identity with both strains, while PXO99^A^ and MAFF311018 share 99% identity. Furthermore, AXO1947 chromosome alignments at the nucleotide level with genomes of strains PXO99^A^ and MAFF311018 reveal large chromosome inversions and rearrangements. Data mining indicates that AXO1947 contains nine TAL (transcriptional activator-like) effector genes. One TAL effector gene is identical to TalC, which induces the rice nodulin-3 *Os11N3* (*OsSWEET14*) (14).

### Nucleotide sequence accession number.

The final closed version of the *X. oryzae* pv. oryzae strain AXO1947 genome has been deposited at GenBank under the accession number CP013666.
